# Clinical implication of electrocardiogram change in patients experiencing lung transplantation with end stage lung disease

**DOI:** 10.3389/fphys.2024.1440307

**Published:** 2024-10-29

**Authors:** Ah Young Leem, Hee Tae Yu, MinDong Sung, Kyung Soo Chung, Yeonkyeong Kim, Ala Woo, Song Yee Kim, Moo Suk Park, Young Sam Kim, Young Ho Yang, Ha Eun Kim, Jin Gu Lee, Kyuseok Kim, Kyu Bom Kim, Boyoung Joung, Junbeom Park, Su Hwan Lee

**Affiliations:** ^1^ Division of Pulmonary and Critical Care Medicine, Department of Internal Medicine, Severance Hospital, Yonsei University College of Medicine, Seoul, Republic of Korea; ^2^ Division of Cardiology, Department of Internal Medicine, Severance Cardiovascular Hospital, Yonsei University College of Medicine, Seoul, Republic of Korea; ^3^ Department of Thoracic and Cardiovascular Surgery, Severance Hospital, Yonsei University College of Medicine, Seoul, Republic of Korea; ^4^ Department of Biomedical Engineering, Eulji University, Seoul, Republic of Korea; ^5^ Department of Radiation Convergence Engineering, College of Health Science, Yonsei University, Wonju, Gangwon, Republic of Korea; ^6^ Division of Cardiology, Department of Internal Medicine, College of Medicine, Ewha Womans University, Seoul, Republic of Korea; ^7^ Department of Biomedical Engineering, Emory University School of Medicine, Atlanta, GA, United States

**Keywords:** electrocardiogram, end-stage lung disease, lung transplantation, risk factor, prognosis

## Abstract

**Introduction:**

End-stage lung disease causes cardiac remodeling and induces electrocardiogram (ECG) changes. On the other way, whether lung transplantation (LTx) in end-stage lung disease patients are associated with ECG change is unknown. The object of this study was to investigate ECG changes before and after LTx in end-stage lung disease patients and whether these changes had clinical significance.

**Method:**

This was a single-center retrospective cohort study of 280 end-stage lung disease patients who consecutively underwent LTx at a tertiary referral hospital. ECG findings before LTx and within 1 week and 1, 3, and 6 months after LTx were obtained and analyzed. To find clinical meaning, the ECG at 1 month after LTx was analyzed according to 1-year survival (survivor vs non-survivor groups). Survival data were estimated using the Kaplan–Meier method.

**Results:**

Significant differences were observed in the PR interval, QRS duration, QT interval, QTc interval, and heart rate before LTx and 1 month after LTx; the PR interval, QRS duration, QTc interval, and heart rate were decreased. Particularly, the QTc interval was significantly decreased 1 month after LTx, whereas there was no significant change in the QTc interval from 1 to 6 months thereafter. The PR interval, QT interval, QTc interval, and heart rate were significantly different between the survivor and non-survivor groups. The serial changes in QTc interval before LTx and 1 and 3 months after LTx were also significantly different between the survivor and non-survivor groups (*p* = 0.040 after adjusting for age and body mass index). Upon dividing the patients based on the range of QTc interval change ≤ -8 ms, >-8–10 ms, >10–35 ms, >35 ms), the survival rate was significantly lower in the group whose QTc interval at 1 month after LTx decreased by > 35 m (*p* = 0.019).

**Conclusion:**

LTx in patients with end-stage lung disease may induce ECG changes. Patients whose QTc interval at 1 month after LTx decreased by > 35 ms have a significantly higher 1-year mortality rate. Hence, these ECG changes may have clinical and prognostic significance.

## Introduction

End-stage lung disease is a severe disease that causes cardiac remodeling, and lung transplantation (LTx) can also induce marked changes to the heart (Panagiotou et al., 2017). The heart is connected to pulmonary vessels within the thorax, and the condition of the lung and pulmonary vessels can affect the heart. Hyperexpanded lungs compress the heart and diaphragm, which may cause clockwise rotation of the heart location. Chronic hypoxemia due to chronic lung disease also causes pulmonary vasoconstriction with elevation of pulmonary artery pressure. Destruction of lung tissues causes pulmonary capillary injury and increases the resistance of pulmonary vasculature, and increased pulmonary arterial pressures cause right atrial and ventricular afterload (Pinsky, 2018; Harrigan and Jones, 2002). Furthermore, changes in intrathoracic pressure affect the systemic venous return and left ventricular systolic pressure (Lalande et al., 2012).

The 12-lead electrocardiogram (ECG) provides several information regarding cardiac electric activity (Stracina et al., 2022). Abnormalities in heart function affect ECG findings, and thus, the ECG plays an important role in the diagnosis of arrhythmias and several cardiovascular diseases such as myocardial infarction, myocarditis, pericarditis, myocardial fibrosis, and inherited defects (Yu et al., 2021; Van Mieghem et al., 2004). Hence, cardiac changes related to lung disease can affect ECG findings. Some patients with chronic lung disease or pulmonary thromboembolism show abnormal ECG (Pinsky, 2018; Van Mieghem et al., 2004). Several studies have shown ECG abnormalities in end-stage heart failure and confirmed ECG changes after heart transplantation, and these ECG changes are clinically helpful (Hashim et al., 2022; Babuty et al., 1993). Moreover, electrolyte imbalances also affect ECG findings. In patients with end-stage kidney disease with electrolyte abnormalities, ECG changes are observed after kidney transplantation (Johri et al., 2009; Lee et al., 2021).

The ECG may also change in patients with end-stage lung disease who undergo LTx. Some studies reported arrhythmias after LTx (Orrego et al., 2014; Kim et al., 2020), and one study reported that bilateral LTx reduced heart rate variability and complexity of cardiac autonomic modulation (Tobaldini et al., 2021). However, data on changes in individual ECG parameters such as PR interval, QT interval, and QTc interval after LTx are scarce. Therefore, in this study, we aimed to investigate ECG changes before and after LTx in end-stage lung disease patients and whether these changes had clinical significance. We hypothesized that there are cardiac changes after LTx, and these changes can be reflected in the ECG.

## Materials and methods

### Study design and population

**FIGURE 1:**
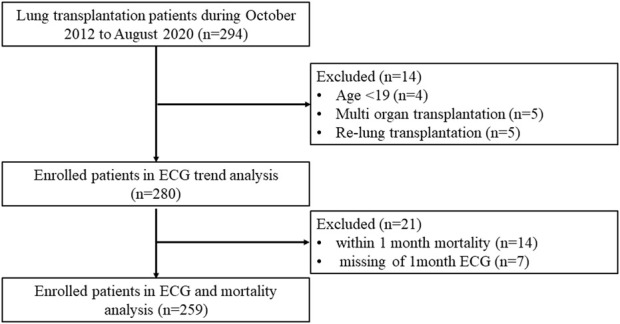
Study flow.

This was a single-center retrospective cohort study of patients with end-stage lung disease who underwent LTx at Severance Hospital in South Korea between October 2012 and August 2020. Among the 294 patients identified, 14 were excluded due to young age (<19 years, n = 4), re-transplantation (n = 5), and multi-organ transplantation (n = 5). Finally, 280 patients were evaluated (Figure 1). To find clinical meaning, the ECG findings at 1 month after LTx was analyzed according to 1-year survival; the patients were accordingly divided into the survivor and non-survivor groups.

All LTx procedures were performed under intraoperative extracorporeal membrane oxygenation or cardiopulmonary bypass, and all patients received induction immunosuppression with methylprednisolone 250 mg intraoperatively and 0.5 mg/kg/day for 3 days postoperatively. After LTx, most patients received a triple immunosuppression regimen comprising prednisolone, tacrolimus, and mycophenolate mofetil. Prophylaxis against cytomegalovirus and invasive aspergillosis was provided until 6 months after LTx using valganciclovir and voriconazole or itraconazole. Life-long prophylaxis for *Pneumocystis jirovecii* pneumonia was provided using trimethoprim/sulfamethoxazole.

Patient data were obtained from the electronic medical records of the hospital. Clinicodemographic data including age, sex, cause of LTx, presence of hypertension and diabetes mellitus, and survival or death were evaluated.

The LTx protocol includes hospitalization for routine check-ups at 1, 3, 6, and 12 months after surgery, and ECGs were taken at these times. Therefore, ECG findings before LTx and within 1 week after LTx and at 1, 3, and 6 months after LTx were obtained.

### ECG protocol and parameter definition

All patients underwent a regular 12-lead ECG check-up and 2-dimensional transthoracic echocardiography (TTE) before and after LTx. Data on the following ECG parameters were obtained: PR interval; QRS duration; QT interval; QTc interval; and P, R, and T axes. The PR interval extended from the beginning of the P wave until the beginning of the QRS complex. The QRS duration was defined as the time from the first downward deflection after the P wave to the second downward deflection after the R wave. The QT interval was measured from the onset of the QRS complex to end of the T wave that returned to the TP baseline. Every QT interval was corrected for the patient heart rate using Bazett’s formula: QTc = QT√RR (in ms) (Page et al., 2016). The normal range of the QTc interval was as 350–450 m for males and 360–460 m for females (Rezuş et al., 2015). On TTE, we acquired the ejection fraction (measured by M-mode), right ventricular systolic pressure (RVSP), tissue Doppler imaging (TDI), peak systolic velocities of tricuspid annulus derived from TDI (TDIS), left ventricle end diastolic dimension (LVEDD), left ventricle end‐systolic dimension (LVESD), (measured by Modified Simpson’s method), and E/E′ ratio (E: mitral peak velocity of early filling, E’: early diastolic mitral annular velocity).

### Ethical statement

This study was approved by the Institutional Review Board of Severance Hospital (IRB No. 4–2022–0122) and was conducted according to the principles set forth in the Declaration of Helsinki. The requirement for obtaining informed consent was waived owing to the retrospective nature of the study.

### Statistical analyses

Continuous variables did not satisfy normality, so they were analyzed using the Mann-Whitney U test and expressed as medians with interquartile ranges (IQRs). Meanwhile, categorical variables are expressed as numbers with corresponding percentages and were analyzed using chi-squared or Fisher’s exact tests. Owing to non-normal distribution of the data, ECG findings before and after LTx were analyzed using Wilcoxon signed rank test. Serial ECG findings at different time points were compared between the two groups using generalized estimating equation (GEE) to estimate the parameters of a generalized linear model with a possible unmeasured correlation. Survival data were estimated using the Kaplan–Meier method, and significant differences between the two groups were determined using the log-rank test. All statistical analyses were performed using SPSS version 26.0 (IBM, Armonk, NY), and *p* < 0.05 was set to indicate statistical significance.

## Results

### Baseline patient characteristics

The median patient age was 57 (IQR: 48.3–63) years, and 64.3% (n = 180) of the patients were male. Idiopathic pulmonary fibrosis (n = 151, 53.9%) was a major cause of LTx, and almost all patients (n = 269, 96.1%) underwent bilateral LTx. In the preoperative TTE, the mean right ventricular systolic pressure was 48.5 (IQR, 38–79.9) mmHg. For the preoperative ECG, 37.5% (n = 105) of the patients showed abnormal QTc interval. Table 1 shows the other baseline patient characteristics.

**TABLE 1:** Baseline patient characteristics (N = 280).

Characteristic	Value
Age (years)	57 (48.3–63)
Sex	
Male	180 (64.3)
Female	100 (35.7)
Body mass index	20.9 (18.4–23.6)
Indication for lung transplantation	
IPF	151 (53.9)
CTD-ILD	48 (17.1)
Bronchiectasis	19 (6.8)
LAM	5 (1.8)
COPD	10 (3.6)
BO	19 (6.8)
Pulmonary hypertension	6 (2.1)
Others	22 (7.9)
Hypertension	67 (23.9)
Diabetes mellitus	80 (28.6)
Lung transplantation	
Bilateral	269 (96.1)
Single	11 (3.9)
One-year mortality	94 (32.1)
Sepsis	58 (61.7)
Cardiac problem	17 (18.1)
Hemorrhage	4 (4.3)
Others	15 (16)
Preoperative TTE parameters	
EF (%)	64 (58–69)
E/E′	10 (8–12)
RVSP mmHg	48.5 (38–79.9)
TDIS	11 (9–13)
LVEDD (mm)	42 (39–46)
LVESD (mm)	29 (25–31)
Preoperative ECG parameters	
PR interval (ms)	144 (130–164)
QRS duration (ms)	86 (78–96)
QT interval (ms)	361 (336–392)
QTc interval (ms)	445 (432–464)
Heart rate (/min)	95 (83–106)
Atrial fibrillation	2 (0.71)
Abnormal QTc^a^	105 (37.5)

Values are expressed as n (%) or the median (interquartile range) unless otherwise indicated.

^a^ The normal range for the QTc, interval is set at 350–450 m for males and at 360–460 m for females.

Abbreviations: IPF, idiopathic pulmonary fibrosis; CTD-ILD, connective tissue disease-associated interstitial lung disease; LAM, lymphangioleiomyomatosis; COPD, chronic obstructive pulmonary disease; BO, bronchiolitis obliterans; TTE, transthoracic echocardiography; EF, ejected fraction; E/E′, E: mitral peak velocity of early filling, E’: early diastolic mitral annular velocity; RVSP, right ventricular systolic pressure; TDI, tissue Doppler imaging; TDIS, peak systolic velocities of tricuspid annulus derived from TDI; LVEDD, left ventricle end-diastolic dimension; LVESD, left ventricle end-systolic dimension.

### Changes in ECG and TTE findings

**FIGURE 2:**
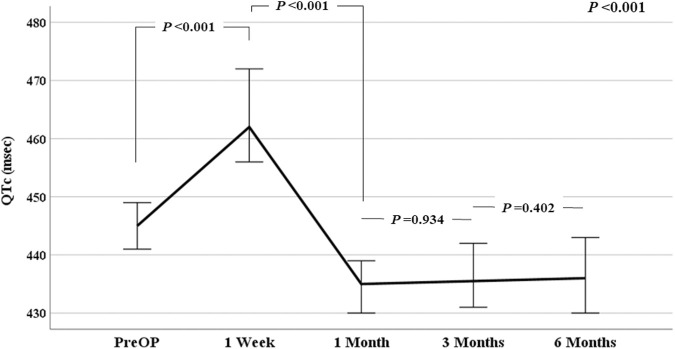
Trend in QTc interval after lung transplantation.

Table 2 shows the ECG findings before LTx and at 1, 3, and 6 months after LTx. The PR interval, QRS duration, QT interval, and QTc interval were significantly different before LTx and 1 month after LTx (all *p* < 0.001). The PR interval, QRS duration, QTc interval, and heart rate were all decreased after LTx than before LTx. The QTc interval was significantly decreased 1 month after LTx, although no significant change was observed from 1 month to 6 months thereafter. Figure 2 shows the trend in QTc interval after LTx; the QTc interval was consistently reduced after LTx (*p* < 0.001). Additionally, we conducted further analysis to explore potential differences based on the type of terminal lung disease and the type of lung transplantation (bilateral vs single). There were no significant differences in the trend of QTc interval changes after LTx between idiopathic pulmonary fibrosis and other lung diseases (*p* = 0.514). Furthermore, we found no differences in the trend of QTc changes after LTx between bilateral and single lung transplantation (*p* = 0.445).

**TABLE 2:** Change in ECG parameters after lung transplantation.

	Before LTx	1 month after LTx	*Before* vs. *1month* *p*-value	3 months after LTx	*1 month* vs. *3months* *p*-value	6 months after LTx	*3months* vs. *6months* *p-*value
PR interval (ms)	144 (130–164)	140 (126–158)	<0.001	138 (126–154)	<0.001	138 (128–154)	0.001
QRS duration (ms)	86 (78–96)	84 (74–92)	<0.001	84 (78–92)	0.002	84 (78–94)	0.612
QT interval (ms)	361 (336–392)	351 (332–372.5)	<0.001	364 (342–384)	<0.001	364 (342–384)	0.25
QTc interval (ms)	445 (432–464)	435 (413–454)	<0.001	435.5 (417–455.3)	0.813	436 (417–458)	0.43
Heart rate (/min)	95 (83–106)	94 (84–106)	0.713	87 (77–99)	<0.001	87 (78–102)	0.049
P axis (°)	45 (32.8–61)	50 (33.5–64)	0.115	52 (35–67)	0.09	52 (35.8–66)	0.327
R axis (°)	46 (1–88)	50 (17–76)	0.496	47 (13–70)	0.01	43 (11.8–71)	0.007
T axis (°)	26 (6–52)	58 (33–82)	<0.001	60 (37–72)	0.18	58 (39–74)	0.437
Atrial fibrillation (n,%)	2 (0.7)	5 (1.9)		3 (1.18)		1 (0.45)	

Values are expressed as the median (interquartile range) unless otherwise indicated.

LTx, lung transplantation.

Among the 259 patients who survived at 1 month and underwent ECG, 166 (64.1%) showed decreased QTc interval. Although the QTc interval of the 93 patients (35.9%) was increased, the interval was within the normal range in 47 patients. At 1 month after LTx, 71 patients (27.4% 71/259) showed abnormal QTc interval. Although the P- and R-axes were not significantly different before and after LTx, the T-axis was significantly increased after LTx (*p* < 0.001). There was no increase in the prevalence of atrial fibrillation during the follow-up period. In the TTE findings before and after LTx, there were no significant differences in EF, TDIS, LVEDD, or LVESD. However, RVSP significantly decreased from a median of 54 (40–66) before LTx to a median of 31.5 (26–43) after LTx (*p* < 0.001).

### ECG findings and 1-year mortality

The survivor group was significantly younger (55 years vs. 62 years, *p <* 0.001) and had significantly lower BMI (20.7 kg/m^2^ vs. 21.3 kg/m^2^, *p* = 0.045) than the non-survivor group. For ECG findings, the PR interval, QT interval, QTc interval, and heart rate were significantly different between the two groups; the QTc interval was significantly higher in the survivor group (437 m vs 421 m, *p* = 0.005). However, the proportion of patients with abnormal QTc interval was not significantly different between the two groups (29.2% vs 23.5%, *p* = 0.209). There were also no significant differences in TTE parameters at the preoperative period and 1 month after LTx between survivor and non survivors (Table 3). Additionally, Cox proportional hazards regression analysis, including variables such as age, sex, BMI and ECG parameters, was conducted to gain a deeper understanding of the associations between ECG changes and outcomes. In the multivariate analysis, age, QT interval, and QTc interval from the 1-month ECG after LTx were significantly associated with 1-year mortality (Table 4).

**TABLE 3:** Comparison of patient characteristics according to survival at 1 year after lung transplantation.

	Survivors (n = 178)	Non-survivors (n = 81)	*p*-value
Age (years)	55 (44–61)	62 (56.5–66)	<0.001
Male sex	118 (66.3)	60 (33.7)	0.17
Body mass index	20.7 (18.3–23.4)	21.3 (19.0–25.0)	0.045
Hypertension	43 (24.2)	21 (25.9)	0.436
Diabetes mellitus	50 (28.1)	27 (33.3)	0.238
1-Month ECG parameters			
PR interval (ms)	138 (125.5–152)	147 (130–170)	0.016
QRS duration (ms)	84 (74–94)	82 (75–89)	0.072
QT interval (ms)	354 (335.5–374.5)	334 (314–359)	<0.001
QTc interval (ms)	437 (420–456)	421 (400–450)	0.005
Heart rate (/min)	93 (83–103)	98 (89–114)	0.001
abnormal QTc^a^	52 (29.2)	19 (23.5)	0.209
QTc interval according to cause of death			
Sepsis (n = 55)		419 (397–451)	
Cardiac problem (n = 11)		440 (425–443)	
Hemorrhage (n = 3)		409 (401–409)	
Others (n = 12)		417.5 (407.3–455.5)	
Preoperative TTE parameters			
EF (%)	64.5 (59–69) N = 172	64 (57–69) N = 78	0.363
E/E′	10 (8.3–12) N = 129	11 (9–13) N = 63	0.270
RVSP (mmHg)	50 (39–60.3) N = 147	48 (38–65.5) N = 73	0.700
TDIS	11 (9–13) N = 134	11 (9–13.8) N = 57	0.717
LVEDD (mm)	42 (39–47) N = 162	42 (38–46) N = 75	0.510
LVESD (mm)	29 (25–32) N = 162	28 (24–31) N = 75	0.442
TTE parameters 1-month postoperatively			
EF (%)	64 (58–68) N = 167	63 (59–70) N = 78	0.553
E/E′	10 (7–12) N = 86	11 (8.3–13.8) N = 32	0.184
RVSP (mmHg)	30 (26–37) N = 113	32.5 (25–37.3) N = 54	0.501
TDIS	11 (9–13) N = 127	11 (9.8–13) N = 59	0.706
LVEDD (mm)	42 (39–46) N = 154	41 (38.5–45) N = 73	0.165
LVESD (mm)	29 (25–32) N = 154	28 (25–30) N = 73	0.163
Number of drugs that cause QTc prolongation^b^	1 (0–2)	1 (0–2)	0.829

Values are expressed as n (%) or the median (interquartile range) unless otherwise indicated.

^a^ The normal range of QTc, interval is defined as 350–450 m for males and as 360–460 m for females.

^b^ Drugs that cause QTc, prolongation: trazodone, itopride, atenolol, linezolid, meropenem, mosapride, ondansetron, ciprofloxacin, levofloxacin, haloperidol, quetiapine, isoniazid, ramosetron, clonazepam, amiodarone, and escitalopram.

Abbreviations: ECG, electrocardiogram; TTE, transthoracic echocardiography; EF, ejected fraction; E/E′, E: mitral peak velocity of early filling, E’: early diastolic mitral annular velocity; RVSP, right ventricular systolic pressure; TDI, tissue Doppler imaging; TDIS, peak systolic velocities of tricuspid annulus derived from TDI; LVEDD, left ventricle end-diastolic dimension; LVESD, left ventricle end-systolic dimension.

**TABLE 4:** Cox proportional hazards model of the 1-month ECG after lung transplantation and 1-year mortality.

	Univariate		Multivariate	
	HR (95% CI)	*p*-value	HR (95% CI)	*p*-value
Age	1.063 (1.041–1.086)	<0.001	1.062 (1.042–1.084)	<0.001
Female sex	1.247 (0.842–1.847)	0.271		
BMI	1.026 (0.981–1.074)	0.266		
1-Month ECG parameters				
PR interval (ms)	1.003 (0.996–1.010)	0.371		
QRS duration (ms)	0.997 (0.984–1.010)	0.639		
QT interval (ms)	0.988 (0.959–1.018)	0.430	0.983 (0.977–0.989)	<0.001
QTc interval (ms)	1.009 (0.985–1.032)	0.478	1.008 (1.001–1.015)	0.031
Heart rate (/min)	1.006 (0.959–1.056)	0.797		
abnormal QTc^a^	0.738 (0.400–1.360)	0.330		
preQTc minus QTc after 1month LTx	1.004 (1.000–1.008)	0.059	1.004 (1.000–1.008)	0.080

^a^ The normal range of QTc, interval is defined as 350–450 m for males and as 360–460 m for females. Abbreviations: HR, hazard ratio; CI, confidence interval; BMI, body mass index; ECG, electrocardiogram; LTx, lung transplantation.

### Serial change of QTc interval after LTx

**FIGURE 3:**
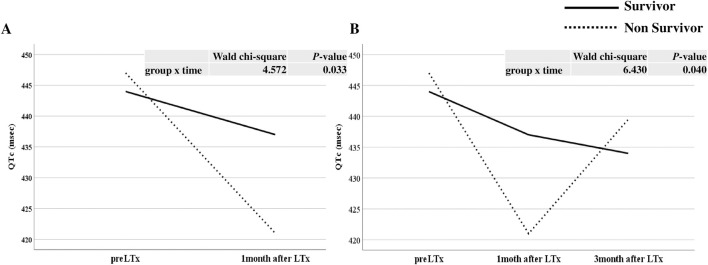
The serial change in QTc interval before and after lung transplantation (LTx) between survivor and non survivor. **(A)** Comparison of changes before LTx and one month after LTx, **(B)** Comparison of changes before LTx, one month after LTx, and three months after LTx.

As shown in Figure 3A, the serial change in QTc interval before LTx and 1 month after LTx was significantly different between the survivor and non-survivor groups after adjusting for age and BMI (*p* = 0.033). The serial changes in QTc interval before LTx and at 1 and 3 months after LTx was also significantly different between the two groups after adjusting for age and BMI (*p* = 0.040, Figure 3B). The non-survivor group showed a more dynamic change in QTc than the survivor group. The number of drugs causing QTc prolongation such as trazodone, itopride, atenolol, linezolid, meropenem, mosapride, ondansetron, ciprofloxacin, levofloxacin, haloperidol, quetiapine, isoniazid, ramosetron, clonazepam, amiodarone, and escitalopram at 1 month after LTx were not significantly different between the two groups.

### Survival impact of QTc change

**FIGURE 4:**
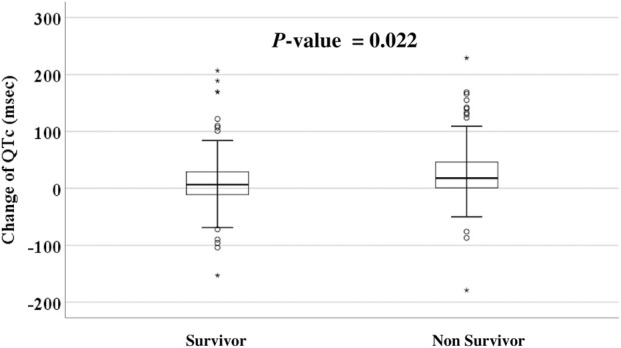
QTc interval change between survivor and non-survivor.

**FIGURE 5:**
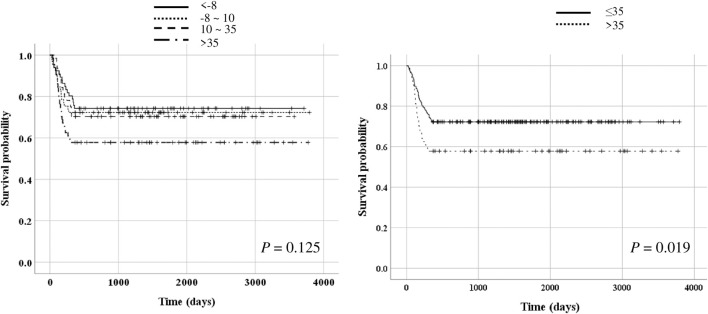
Survival impact of QTc interval change.

Given the findings mentioned earlier, additional analysis was conducted to determine whether the change in the range of QTc interval after LTx was associated with prognosis. The median difference in QTc interval before LTx and 1 month after LTx was 10 (IQR, -8–35 m). There was a significant difference in the change of QTc interval before LTx and 1 month after LTx between survivors and non-survivors (6.5 [-11–29]ms vs 18 [0.5–46]ms, *p* = 0.022, Figure 4). The patients were then divided into four groups according to the interquartile range of QTc interval change (≤-8 ms, -8–10 m, 10–35 m, >35 m). Kaplan–Meier survival analysis showed that the survival rate was significantly lower in the group whose QTc interval at 1 month after LTx was decreased by > 35 m (*p* = 0.019, Figure 5). Table 5 shows the comparison of characteristics between patients whose QTc interval before LTx was changed by ≤ 35 m and >35 m 1 month after LTx. The group with a >35 m change had a significantly higher 1-year mortality rate (27.3% vs 43.1%, *p* = 0.021). Regarding the cause of death, the rate of sepsis was also significantly higher in this group (17.5% vs 32.3%, *p* = 0.022). There was no significant difference in other variables such as age, sex, and comorbidities.

**TABLE 5:** Comparison of patient characteristics according to change in QTc interval from pre-to post-lung transplantation.

	QTc <35 (N = 194)	QTc >35 (N = 65)	*p*-value
Age (years)	58 (49.8–63)	58 (46.5–63)	0.902
Male sex	125 (64.4)	41 (63.1)	0.882
Body mass index	21.1 (18.6–23.6)	20.3 (17.7–23.4)	0.408
Hypertension	48 (24.78)	16 (24.6)	1.000
Diabetes mellitus	62 (32)	15 (23.1)	0.210
1-Month ECG parameters			
PR interval (ms)	138 (126–156)	146 (130.1–160)	0.124
QRS duration (ms)	84 (74–92)	82 (74–92)	0.590
QT interval (ms)	354 (334–374)	339 (309–368)	0.004
QTc interval (ms)	440 (425–460.3)	411.5 (392.8–427)	<0.001
Heart rate (/min)	96 (86–106)	88 (81.5–103)	0.051
abnormal QTc^a^	62 (32)	9 (13.8)	0.006
One-year mortality	53 (27.3)	28 (43.1)	0.021
Cause of death			0.022
Sepsis	34 (17.5)	21 (32.3)	
Cardiac problem	10 (5.2)	1 (1.5)	
Hemorrhage	1 (0.5)	2 (3.1)	
Others	8 (4.1)	4 (6.2)	
Preoperative TTE parameters			
EF (%)	64 (59–69) n = 187	61 (57–69) n = 63	0.096
E/E′	10 (9–12) n = 145	9 (8–12) n = 47	0.331
RVSP (mmHg)	48.5 (37.8–60.3) n = 162	50.5 (41.5–67.3) n = 58	0.315
TDIS	12 (10–13.8) n = 144	10 (8–12) n = 47	<0.001
LVEDD (mm)	42 (39–47) n = 178	42 (38–46) n = 60	0.208
LVESD (mm)	29 (25–31.5) n = 177	29 (25–32) n = 60	0.738
TTE parameters 1-month postoperatively			
EF (%)	64 (59–68) n = 183	61 (58–68) n = 62	0.193
E/E′	10 (8–12) n = 93	10 (6–11.5) n = 25	0.213
RVSP (mmHg)	31 (26–37) n = 121	30.5 (26–36) n = 46	0.509
TDIS	11 (9–13) n = 127	11 (9.8–13) n = 59	0.355
LVEDD (mm)	42 (39–46) n = 171	42 (40–45) n = 56	0.842
LVESD (mm)	28 (25–31) n = 171	29 (25.2–31.8) n = 56	0.645
Number of drugs that cause QTc prolongation^b^	1 (0–2)	1 (0–1)	0.975

Values are expressed as n (%) or median (interquartile range) unless otherwise indicated.

^a^ The normal range of QTc, interval is defined as 350–450 m for males and 360–460 m for females.

^b^ Drugs that cause QTc, prolongation: trazodone, itopride, atenolol, linezolid, meropenem, mosapride, ondansetron, ciprofloxacin, levofloxacin, haloperidol, quetiapine, isoniazid, ramosetron, clonazepam, amiodarone, and escitalopram.

ECG, electrocardiogram; TTE., transthoracic echocardiography; EF, ejected fraction; E/E′, E: mitral peak velocity of early filling, E’: early diastolic mitral annular velocity; RVSP, right ventricular systolic pressure; TDI, tissue doppler imaging; TDIS, peak systolic velocities of tricuspid annulus derived from TDI; LVEDD, left ventricle end-diastolic dimension; LVESD, left ventricle end-systolic dimension.

## Discussion

### Main findings

In this study, we investigated ECG changes before and after LTx in end-stage lung disease patients and whether these changes had clinical significance. The current study found that ECG parameters were changed after LTx in end-stage lung disease patients. The QTc interval decreased 1 month after LTx and remained lower than that before LTx. Furthermore, the change in QTc interval was associated with prognosis after LTx. Patients whose QTc interval was decreased by > 35 m at 1 month after LTx showed poorer prognosis than those whose QTc interval decreased by ≤ 35 m at 1 month.

### Association between lungs and heart

The heart and lungs are closely connected; therefore, in patients with chronic lung disease, anatomical remodeling in the heart occurs (Panagiotou et al., 2017). This remodeling can be diagnosed through anatomical alterations that may present as hypertrophy or enlargement of the right ventricle or can be confirmed through hypertrophy of the left ventricle or enlargement of the atrium (Azevedo et al., 2016). However, our data showed that RVSP was significantly reduced but there were no differences in cardiac ejection fraction, left ventricular size, or right ventricular function.

The occurrence of these anatomical changes in the heart depends on the type of lung diseases that cause hemodynamic changes and varies depending on the duration of the disease (Han et al., 2007). Therefore, predicting the severity or duration of lung disease based on these anatomical changes of the heart is challenging. Our analysis showed that electrophysiological changes on the ECG might precede these anatomical changes. The T axis is related to ventricular repolarization (Kors et al., 1998), and the current study reports significant changes in the T axis on ECG 1 month after LTx. The QTc interval is related to repolarization of the cardiac muscle cells (Sarazan, 2014). In the current study, all QRS durations on the ECG were within the normal range of ≤120 m. While the QTc interval was maintained within the normal range, the change was sensitive to changes before and after LTx. The QTc interval tended to temporarily increase immediately after LTx but then decrease 1 month after LTx and remained stable thereafter. However, patients with large changes before and after surgery (>35 m) had poor prognosis after LTx.

### Pre-sepsis left ventricular change

There are two crucial points to consider regarding our results. The first pertains to the electrical remodeling of the heart, indicated by the QTc interval, which sensitively mirrors hemodynamic changes due to chronic lung disease and transplantation. As previously mentioned, anatomical changes in the heart resulting from chronic lung disease necessitate considerable time and energy; thus, these changes are not as responsive as electrical remodeling. Conversely, notable alterations in the electrical remodeling of the heart signify a substantial impact of transplantation on chronic lung disease. The patients in this study showed a decreased in QTc interval after LTx, which may reflect the cardiac remodeling caused by the lung disease prior to transplantation, potentially influencing the postoperative prognosis. The second point of consideration is that the primary cause of patient mortality was sepsis rather than deteriorating cardiovascular disease. Sepsis-induced alterations in blood oxygen saturation and ion concentrations (Na, K, Ca, and Mg) may have impacted the electrical remodeling, including repolarization of the heart muscle cells. In general, the QTc interval exhibits wide variations not only because of changes in blood ion concentration but also owing to the administration of antibiotics and other medications. Consequently, we conducted an analysis of the use of antibiotics and psychiatric drugs known to influence the QTc interval, and confirmed no significant difference between the survivor and non-survivor groups (Table 3). Electrical remodeling of the heart, represented by the QTc interval, sensitively reflects the hemodynamic changes resulting from chronic lung disease. In the current study, no association between change of QTc interval and cardiac disease-related mortality was observed. The cardiac-related mortality rate was 18%, and over 50% of the patients died during the early period after LTx. This result supports that a large change in QTc interval after LTx may indicate vulnerability to sepsis through an indirect correlation with electrical remodeling of the heart rather than direct cardiac problems. Significant QTc interval changes after LTx may be indicative of the disease severity before transplantation. Furthermore, by representing sepsis-induced alterations in ion concentration after transplantation, electrical alteration is presumably a more rapid marker. Although, the observed increase in mortality does not necessarily indicate that patients with prolonged QTc interval are inherently more susceptible to sepsis. However, changes in QTc on an ECG may serve as subtle markers of early septic conditions. Therefore, further research with stronger controls and prospective study designs is needed to clarify the underlying mechanisms.

### Limitations and meaning

To our best knowledge, this is the first study to analyze changes in individual ECG parameters including PR interval, QRS duration, QT interval, QTc interval, and axis after LTx. However, this study is limited by its retrospective single-center design and the insufficient follow-up period, which do not allow for the examination of long-term changes. The results may not be generalizable to other countries due to the study being conducted at a single center and because all enrolled patients are Asian. Furthermore, other factors that may influence ECG changes including post-operative course and several drugs that were not adjusted for, may have been overlooked. Prospective studies in which several factors that can affect the ECG are well controlled are needed to validate our findings. In addition, future studies with larger and more diverse encompassing a wide range of lung diseases, will build upon this knowledge.

## Conclusion

This study found that ECG findings may change after LTx in patients with end-stage lung disease. Particularly, a QTc interval decreased by > 35 m at 1 month after LTx is associated with a significantly higher 1-year mortality rate. Thus, these ECG changes may have clinical and prognostic significance.

## Data Availability

The datasets presented in this article are not readily available because Limited due to IRB policy. Data requests require re-review by the IRB. Requests to access the datasets should be directed to SL, hihogogo@yuhs.ac.

## Abbreviations

ECG, electrocardiogram; IQRs, interquartile ranges; LTx, lung transplantation; TTE, transthoracic echocardiography.
